# Examining GDP Growth and Its Volatility: An Episodic Approach

**DOI:** 10.3390/e23070890

**Published:** 2021-07-13

**Authors:** Jakub Bartak, Łukasz Jabłoński, Agnieszka Jastrzębska

**Affiliations:** 1 Department of Process Management, Cracow University of Economics, ul. Rakowicka 27, 31-510 Kraków, Poland; bartakj@uek.krakow.pl; 2 Department of Macroeconomics, Cracow University of Economics, ul. Rakowicka 27, 31-510 Kraków, Poland; lukaszj@uek.krakow.pl; 3 Faculty of Mathematics and Information Science, Warsaw University of Technology, ul. Koszykowa 75, 00-662 Warsaw, Poland

**Keywords:** economic growth, volatility, episodes, structural breaks, genetic algorithm

## Abstract

In this paper, we study economic growth and its volatility from an episodic perspective. We first demonstrate the ability of the genetic algorithm to detect shifts in the volatility and levels of a given time series. Having shown that it works well, we then use it to detect structural breaks that segment the GDP per capita time series into episodes characterized by different means and volatility of growth rates. We further investigate whether a volatile economy is likely to grow more slowly and analyze the determinants of high/low growth with high/low volatility patterns. The main results indicate a negative relationship between volatility and growth. Moreover, the results suggest that international trade simultaneously promotes growth and increases volatility, human capital promotes growth and stability, and financial development reduces volatility and negatively correlates with growth.

## 1. Introduction

The motivation for studying the relationship between business cycle volatility and economic growth stems from several reasons. First, research in this area can verify whether short-term fluctuation is beneficial or detrimental to long-term economic growth. Therefore, both answers are possible—on the one hand, volatility may reflect “creative destruction” processes and costly adoption of new technology. On the other hand, instability may increase uncertainty and discourage investment, as well as have negative social consequences resulting with the waste of human capital. Given the lack of consensus on theoretical underpinnings, there is a need for empirical studies that could provide valuable insights for discussion. Second, understanding the relationship between volatility and economic growth is important from a practical perspective and is essential for timely and effective economic policy. With positive linkages between the two, a policy-maker who seeks to stabilize the business cycle can simultaneously undermine long-term economic growth. Negative linkages between these phenomena can create circumstances for smoothing business cycle fluctuations that are consistent with the policy objective of high long-run growth [1]. Finally, large cross-country differences in output volatility [2] and shifts in growth stability across countries [3] have been observed. Such differences in the time characteristics of growth time series require explanations, especially regarding growth capacity.

In this paper, we aim to examine the relationship between economic growth and its volatility within an episodic research perspective. We point out the following advantages of using an episodic approach. First, temporal characteristics of the GDP growth time-series call for the analysis focused on episodes; otherwise, these characteristics are ignored. In general, the use of raw, unsegmented GDP time series is subject to the problems identified by Pritchett [2], namely, low statistical power, higher measurement error, higher risk of endogeneity, and dynamic misspecification. These problems particularly apply to high-frequency panel data models [2]. Second, in the specific case of our research task, as we show in the following sections, time series of GDP growth often consist of distinct segments with different levels of volatility and growth rates.

In a traditional research approach, such cases would be treated in one of the following ways: (a) in a cross-sectional setting volatility and growth would be measured over the entire time horizon, meaning that the volatility/growth shift would be completely ignored; (b) in a panel data setting volatility and growth would be measured in non-overlapping time intervals of a predefined length. The disadvantages of this approach, in addition to the above-mentioned fundamental ones raised by Pritchett [2], are that the length of the interval is set arbitrarily and the intervals do not correspond to true changes in growth or volatility. Third, the identification of stable/variable and low/high growth episodes allows to ask questions about the mechanisms underlying the transition between these patterns.

Segmentation of time series is elementary for such studies, as it allows to extract episodes, which in turn provide the basis for interpretation of results. Being aware of the important role of the created episodes, we propose to solve the problem of detecting change points in time series using modern heuristic optimization techniques. In particular, we apply a genetic algorithm (GA) to segment the GDP time series.

Let us state the key novelty introduced in the paper:

- We introduce the episodic research perspective to the studies on growth–volatility relationship. To the best of our knowledge, our study is the first attempt to apply the episodic approach to the analysis of growth and volatility relationship.
- We apply a genetic algorithm to segment the GDP time series. Based on this, we identify growth episodes with different volatility and growth rates. In addition to qualitative analyses, we present a quantitative study demonstrating the effectiveness of the genetic algorithm in the time series segmentation task. Furthermore, we compare episodes obtained using the genetic algorithm with those obtained using the Bai–Perron [4] method, which is considered a classic in this area.
- The ability to identify patterns of (un)stable growth allowed us to investigate their determinants and analyze the growth–variability relationship in a new perspective. In particular, once growth episodes are identified, we can identify the driving forces of economic growth that are crucial in a given episode.

The remainder of the paper is structured as follows. In Section 2, we review the literature on the relationship between economic growth and its volatility. In Section 3, we present the genetic algorithm as a tool for detecting structural breaks in economic growth time series, which is sensitive to changes in the level and volatility of the time series. Then, we discuss our approach to studying the links between growth and volatility, and the driving forces behind high and stable growth patterns. In Section 4, we follow with a description of the data. In Section 5, we demonstrate the versatility of the genetic algorithm to identify turning points in artificial and real data. Then, we present the results of ordered and polynomial regression models to provide insight into the growth–variability relationship and to discern the correlates of stable growth. Finally, in Section 6, we summarize and discuss the results obtained.

## 2. Literature Review

Research on volatility and economic growth began in the mid-20th century. Nevertheless, until the early 1980s, they were investigated separately, meaning that the discussion of volatility was outside the research on economic growth. Specifically, research on volatility was concerned with the short research perspective and research on economic growth with the long research perspective. However, the emergence of the real business cycle approach [5,6] provided a methodological platform for viewing business cycle volatility as an immanent part of the economic growth process [7]. Since then, researchers have analyzed the relationship between volatility and economic growth from different perspectives, with ambiguous and sometimes contradictory results. Note that a mixed picture of the interdependence between these issues emerges from theoretical and empirical studies.

The theoretical literature highlights both positive [8,9] and negative [10,11,12] relationships between volatility and economic growth. The positive interdependence between the two grew out of Schumpeter’s concept of creative destruction [8], who stated that during recessions or economic downturns, old technologies are replaced by new ones, which stimulates productivity and economic growth. Following this argument, Black [13] introduced a risk–return trade-off for technology choice. Specifically, he showed that entrepreneurs choosing higher levels of risk are associated with higher expected returns, but also with a correspondingly higher risk of failure. At the same time, researchers have identified other channels, through which volatility and growth are positively related, such as the “disciplining effect” [14], the “cleaning-up” effect [15], or the “opportunity cost” effect [9]. On the other hand, the negative implications of volatility for economic growth are emphasized, in line with Keynes’ rationale that fluctuations in economic activity increase the level of risk, which in turn reduces investment and output growth. Following this line of reasoning, King et al. [16] integrated endogenous growth theory with business cycle theory and showed that transitory output disturbances determine output growth in the long run. The negative relationship between volatility and growth was explained by Arrow’s “learning-by-doing” effects [10] to incorporate technological change [17] and investment irreversibility [18] into the growth process. More recently, the introduction of credit constraints into the Schumpeterian model has yielded a negative nexus between volatility and economic growth [12].

The empirical literature, while richer than the theoretical one, also provides inconclusive evidence. Consistent with research strategies that treat volatility as one of the independent variables in the economic growth equation, early studies used cross-sectional data that supported the Schumpeterian approach. For example, Kormendi and Meguire [19] found positive relationships between volatility and growth by measuring output volatility by the standard deviation of economic growth for 47 countries, as did Grier and Tullock [20]—for a broader group of countries. However, Ramey and Ramey [21], who produced a kind of benchmark study in this area, found a significantly negative relationship between volatility and growth using panel data for 92 countries. Their findings were supported by Van der Ploeg and Poelhekke [22] and Aghion et al. [23]. On the other hand, Döpke [24] and Norrbin and Yigit [1] checked the results of Ramey and Ramey [21] by applying multiple robustness tests and found them to be sensitive to the choice of periods, the group of countries in the sample, and the estimation method.

In the second type of research strategy, the nexus of volatility and growth is examined using generalized autoregressive conditional heteroscedasticity models (e.g., Caporale and McKiernan [25], Fountas and Karanasos [26]). Such studies have also produced contradictory results. Positive nexus for the US economy was found in [25], while no robust relationship was reported in [26]. Lee [27] extended the GARCH-in-mean technique by the dynamic panel data and questioned the both-direction positive relation between growth and volatility. As a result, he showed that although higher growth is accompanied by higher volatility, there is little evidence of causality running from growth to volatility. Moreover, Fang and Miller [28] extended the GARCH-in-mean model into the exponential specification and showed a positive association between volatility and output growth in a long period time series [12].

The third empirical research strategy is to identify the channels through which volatility and growth are related. Scholars have explored the role of openness to foreign trade, financial integration, and foreign direct investment [29,30]. There are also studies examining the impact of political factors (forms of government, electoral rules, forms of state, number of veto players, and age of democracy) on the growth–volatility relationship [31]. Finally, an important strand of research is devoted to the financial development channel. Initially, Aghion and Banerjee [32] showed that the negative effect of volatility on growth depends on the degree of financial development. Beck et al. [33], in turn, found that financial development, understood as the deepening of private credit, can reduce real (rather than monetary) volatility, especially if stock markets are underdeveloped. After using new measures of financial development, Beck et al. [34] presented evidence that financial intermediation increases economic growth while reducing volatility in the long run. However, these effects become weaker over shorter and more recent time horizons. More recently, Silva et al. [35] found that as financial development improves, growth volatility increases faster than average growth itself.

In sum, the reviewed studies emphasize either nonlinear volatility-growth relationships or the fact that this nexus depends on some kind of “threshold level of factors” [35]. That is, different macroeconomic factors can determine positive or negative volatility-growth relationships, depending on the circumstances, such as the level of economic development, financial deepening, openness to foreign trade, and financial market integration, among others. Thus, a meta-analysis of studies examining the growth-volatility relationship emphasizes that the debate has not been resolved [12].

In this paper, we aim to contribute to the existing literature through an alternative research approach, i.e., analyzing different growth patterns, understood as temporal characteristics of a growth time series consisting of growth rate and growth volatility. Specifically, our aim is to distinguish and investigate the correlates of the following growth patterns: low growth and low volatility, high growth and high volatility, low growth and high volatility, and high growth and low volatility. In this context, our study stands out from other studies in the field because it introduces an episodic approach to the macroeconomic study of GDP growth and its volatility.

To achieve the intended aim, we built upon the previous research on the episodic character of economic growth. In this stream of literature, researchers argue that the growth process in many countries can be described by distinct growth episodes, such as growth stagnation, collapse, or acceleration. The switch between these growth periods is often accompanied by dramatic changes in growth rates. The observed characteristics of growth paths require an analysis focused on episodes of accelerating/decelerating growth, rather than an analysis focused on the determinants of variability in annual (or other periods of constant length) growth rates. Thus, much of the research on economic growth has evolved from a “steady-state” perspective toward an “episodic” perspective. In this line of argument, note that the identification of links between economic growth and its variability with a range of driving forces of economic development provides a fertile basis for designing economic policy recommendations for countries at different growth episodes. In particular, the search for growth drivers associated with lower volatility will help to address the problem of economic policy effectiveness under uncertainty highlighted by Brainard [36]. Therefore, identifying the drivers of economic growth that simultaneously stimulate greater stability may be helpful to policy-makers in designing economic policies with greater efficiency.

Note that the episodic approach to growth research has become popular since Perron [37] suggested that ignoring structural breaks in a long-run trend, when testing the nature of the underlying trend, can generate misleading guidance for both statistical inference and policy strategies. Structural breaks can be interpreted as shocks of both exogenous and policy-induced nature that affect the rate of growth, generating shifts from one regime of economic growth to another [38]. A central task in such a research perspective is the identification of turning points (breakpoints) in growth time series that indicate the starting and ending point of a growth episode.

Growth episodes are typically identified using a variant of the Bai and Perron [4] (BP) technique to detect and test multiple, and *a priori* unknown, breakpoints. The BP technique allows the estimation of multiple break dates without prior knowledge of when these breaks will occur. The notable applications of this method in the economics of growth are the detection of growth accelerations and growth collapses [39], and the measurement of duration of growth spells as the time between two structural breaks [40]. Empirical studies also use a modification of the BP method, adjusted by Antoshin et al. [41] to improve the performance of breakpoint detection in small samples (e.g., Berg et al. [42], Arizala et al. [43]). Another extension of the Bai–Perron test has been proposed by Papell and Prodan [44], who impose sign restrictions in their model to ensure that patterns of interests are correctly identified. This technique aims to detect those structural breaks where BP procedure fails, i.e., mainly in cases where significant deviations from the growth trend follow quickly one after another, such as a sharp recession coming just after a temporary upward break (see Bluhm et al. [45]). Finally, Kar et al. [46] propose an interesting 2-step procedure that integrates the BP approach with filter-based methods. In the first step, the procedure requires searching for candidate breaks with the BP technique. In the second step, instead of testing whether the break is statistically significant (which Kar et al. [46] perceive as too restrictive for relatively small samples of growth data), it is proposed to use filters to select significant breakpoints [47,48].

Unlike existing approaches, we propose to identify structural breaks using the genetic algorithm. A feature of GA that is of great importance in this paper is its ability to detect breaks in the level of growth rate, and to distinguish between periods of volatility and stability. It is worth mentioning that there is a number of methods for detecting breaks in volatility itself [49,50], which have been used to test the “great moderation” hypothesis [51,52]. However, to integrate an episodic growth perspective with volatility research, a procedure is needed to jointly identify breakpoints in levels and volatility.

## 3. Methods

The empirical strategy used in this study consists of three interrelated stages. In the first stage, we segment the growth time series into episodes based on structural breaks, which are identified using the genetic algorithm (GA) and the Bai–Perron (BP) technique [4] as a reference approach. The adopted series processing scheme was inspired by the studies of Davis et al. [53,54], who detected the structural breaks of time series using genetic algorithm. The time series were compared using the Minimum Description Length (MDL) method. MDL enables us to model a piecewise autoregression (AR), by fitting a theoretical model as closely as possible to the actual time series. The nature of the autoregressive functions is employed to determine changes in the data. With different orders and parameters, the function allows us to distinguish segments of the time series [55]. In brief, the entire time series is randomly segmented. Then, in each segment, the parameters of the regression are estimated [54]. The variance of the fitted function is then used, along with the number of breaks, regression order, and data length to calculate the MDL. The smaller this value is, the better the theoretical model fits the actual data. In conjunction with the MDL, GA was used to automatically determine the solution with the minimum MDL values.

GA employs population, called generation, of candidate solutions, which are named as genes. Each candidate solution is described by its chromosome. The gene assemblies form a new generation by means of a crossover or mutation operation applied to the old gene generation. The procedure of giving birth to the new generation is repeated until a termination criterion is reached. Though the termination criterion can be either to produce a stable solution that does not change much from one iteration to the next or to create a predefined number of new generations. GA, like all population-based methods inspired by natural selection, is efficient at finding a sufficiently good solution, which is acceptable for many optimization problems in life sciences and engineering [56].

In our program, each genome is a sequence of integers in the range <−1, maxOrder>. A value of −1 indicates no breakpoint. The remaining values describe the order of the AR model created in the segment bounded by neighboring breakpoints. For the first and last of these, the beginning and end of the segment correspond to the beginning and end of the time series in question, respectively. For example, take a genome where values different from −1 occur at the i-th and j-th positions, and all values between the i-th and j-th positions are −1. Then, on a segment of the time series that begins at the i-th position and ends at the j-1-th position, we will build an AR model with an order equal to the value occurring at the i-th position.

If the last breakpoint is found at the i-th position in a given genome, then the value at that position determines the order of the AR model associated with segment starting at the i-th position and ending at the last point in a given time series.

The value of the MDL index is calculated according to the formula:

(1) $$\begin{array}{cc} \; & MDL(m,\left(\tau_{1},p_{1}\right),\left(\tau_{2},p_{2}\right),...,\left(\tau_{m},p_{m}\right))\\ \; & =log_{2}m+m\cdot log_{2}n+\sum_{j=1}^{m}log_{2}p_{j}+\sum_{j=1}^{m}\frac{p_{j}+1}{2}\cdot log_{2}n_{j}+\sum_{j=1}^{m}\frac{n_{j}}{2}\cdot log_{2}\left(2\pi \sigma_{j}^{2}\right)+\frac{n}{2} \end{array}$$

where: m—number of breakpoints; $\tau_{j}$—index of the k-th breakpoint; $p_{j}$—the order of an AR model describing a segment between the breakpoints with indexes j and i; n— length of a given time series (the number of elements in the time series); $n_{j}$— length of a given segment, and $\sigma_{j}^{2}$ is the variance of the given segment (AR model parameter).

The input of the created program requires setting the following parameters:

- popSize—the number of genomes in the population (the default is 200);
- toNextGen—the number of best genomes that get promoted to the next generation;
- maxOrder—the maximal order of the fitted AR model;
- crossProb—the crossover probability;
- noChangeProb—the probability of leaving a fragment of a chromosome unaltered during the mutation process;
- noBPProb—the probability of changing a fragment of a chromosome to −1 during the mutation process;
- maxIter—the maximal number of new generations that can be created;
- noChangeStop—the maximal number of iterations (new generations) that can be created, for which the best candidate solution does not change.

The flow of the procedure is as follows:

1. Initialization: generate a random set of popSize candidate solutions, set the best candidate solution to NULL, set the counter of iterations performed to 0.
2. For each genome compute the MDL and sort the generation by it in ascending order.
3. Set the best candidate solution to the best value from the sorted list. If this value has not changed during the last noChangeStop iterations, or the number of iterations performed has reached maxIter, terminate the procedure and return the best genome.
4. The next generation will be performed by toNextGen best genomes.
5. Create (popSize—toNextGen) genomes for the next generation by repeating the following steps for each nascent genome:
  (a) With probability of crossProb create a new genome X using a crossover operation. That is, to do so, randomly select the C1 and C2 genomes from the current generation. The kth element in X (each genome is a vector) is equal to the kth element in C1 or C2, the probability of picking one of the two is 50%.
  (b) With probability of (1—crossProb) create a new genome X using a mutation operation. That is, randomly select a genome C. At the beginning X = C. Each element in X remains unchanged with probability noChangeProb or is set to −1 with probability noBPProb. In other cases, the new value at a given position is randomly drawn from the interval [0, maxOrder].
6. Create a new generation consisting of the genomes obtained from steps 4 and 5.
7. Increase the number of iterations performed and proceed to step 2.

In this study, the detection of structural breaks is performed with the use of GA. Consequently, we do not employ an analytical definition of structural breaks. Instead, structural breaks are determined with the use of an iterative procedure that minimizes a certain fitness function.

After identifying structural breaks using the GA and BP algorithms, in the second step, we divide the all of the countries in the sample into subgroups according to the value of the within-episode average growth rate, following the pattern suggested by Raihan et al. [48]. Accordingly, we categorize episodes into the following groups:

(2) $$Y_{i,t}=\left\{\begin{array}{cc} 1\;\;\mathrm{if}\;\;y_{i,t}^{*}\;\leq \;-2 & (\mathrm{growth}\;\mathrm{collapse})\\ 2\;\;\mathrm{if}\;\;-2\;<\;y_{i,t}^{*}\;\leq \;0 & (\mathrm{negative}\;\mathrm{growth})\\ 3\;\;\mathrm{if}\;\;0\;<\;y_{i,t}^{*}\;\leq \;2 & \left(\mathrm{stagnation}\right)\\ 4\;\;\mathrm{if}\;\;2\;<\;y_{i,t}^{*}\;\leq \;4 & (\mathrm{moderate}\;\mathrm{growth})\\ 5\;\;\mathrm{if}\;\;4\;<\;y_{i,t}^{*}\;\leq \;6 & (\mathrm{rapid}\;\mathrm{growth})\\ 6\;\;\mathrm{if}\;\;6\;<\;y_{i,t}^{*}\; & (\mathrm{miracle}\;\mathrm{growth}) \end{array}\right.$$

Categories of episodes, elicited with the above procedures, served as a dependent ordinal variable $y_{i,t}$, with levels k=1,…,6; in the ordinal logistic regression. Note that the use of logistic regression is a popular research strategy not only in the episodic approach to economic growth [57]. It is also popular in financial studies addressing the topic of assets prices volatility and risk management (see, e.g., in [58]).

The ordinary logistic regression is given by Equation (3).

(3) $$y_{i,t}=\alpha X_{i,t-1}+\epsilon_{i,t}$$

where α denotes the parameter vector, *X* represents the vector of exogenous variables, and $epsilon_{i,t}$ denotes the residuals at the episode-time level.

The cumulative probability of being in at most *k* categories of growth, is given in Equation (4).

(4) $$Prob\left(y_{i,t}\leq k|X_{i,t-1}\right)=F\left(\zeta_{k}-\alpha X_{i,t-1}\right)$$

where F(·) is the logistic function and ζ is the threshold to be estimated in the model.

Note that the cumulative probabilities for each growth category vary across categories only by a factor of ζ, and the factor α for the independent variable is constant. Therefore, α coefficient obtained in such a model indicates a relationship between the exogenous variable and the odds of being on a more prosperous growth path. Consequently, this settlement should be treated as a simplifying assumption. For example, one cannot rule out the possibility that volatility is associated with sharp growth collapses, but does not affect countries trying to move from a moderate growth path to a rapid growth path. We look at this problem using a set of binary logistic regressions. Instead of the ordered dependent variable defined in Equation (2), we use binary variables taking the value 1, if the growth rate is (a) higher than −2%, (b) higher than 0%, (c) higher than 2%, (d) higher than 4%, or (e) higher than 6%.

Among the set of independent variables, we include a measure of volatility, i.e., the standard deviation within an episode. Note that episodes are recognized directly from the data using the GA and BP approaches. This data-driven approach complements previous studies, in which growth volatility is assessed by standard deviation within a rolling window (with a predefined window size) or as standard deviation over non-overlapping time intervals or over the entire time horizon [35].

In the third step of our analysis, we cluster the growth model based on the within-episode standard deviation and within-episode average growth rate. The identification of clusters is done using the k-means method. The resulting clusters serve as the dependent variable in the multinomial regression models. Thus, we proceed to the analysis of the common determinants of both economic growth and its volatility. Specifically, we estimate the parameters of the model given by Equation (5).

(5) $$\frac{Prob(y_{i,t}=j)}{Prob(y_{i,t}=k)}=\alpha X_{i,t-1}+\epsilon_{i,t}$$

where $y_{i,t}$ is a growth cluster of *k* unordered levels of episode *i* in year *t*, α denotes the parameter vector, *X* represents the vector of exogenous variables, and $\epsilon_{i,t}$ denotes the residuals at the episode-time level. The model parameters given by Equation (5) allow us to infer the correlates of cluster membership, and thus allow us to assess the relationship between the independent variables and growth-stability patterns.

## 4. Data

The study employs an unbalanced panel with annual observations for 182 countries over the period 1951–2017. The numerical values of the variables used are taken from several databases that, on the one hand, provide a reliable and high quality and, on the other hand, a complete set of information about the data reflecting the problem under consideration. In particular, the economic growth rate for the countries in the sample is calculated as the difference of the natural logarithms of the per capita variable (real GDP at constant 2011 prices, i.e., the variable *rgdpna*). The numerical values of *rgdpna*, are taken from the Penn World Table (PWT) database version 9.1 [59]. The same data source is used to calculate the standard deviation of the economic growth rate, which in turn reflects the volatility of economic growth as the main category examined in this study.

In our study, we use several control variables reflecting macroeconomic categories central to the growth-volatility relationship as well as to the growth process itself [14,29,30].

Following the arguments put forward by Aghion et al. [23], Beck et al. [34], Silva et al. [35], among others, we use a measure of financial development (*FD*) to capture the impact of international financial markets on the growth–volatility relationship. It is worth emphasizing that, as highlighted by, e.g., the authors of [60,61], the nature of international financial markets as well as the financial development process itself has changed since the end of the Great Moderation. Therefore, we include in the control set a measure of financial development that is taken from [62].

Moreover, as indicated in the literature, e.g., in [30,63], our study uses a measure of openness, defined as the sum of the share of exports and imports of goods at current PPP. Simultaneously, in order to capture the drivers of the economic growth process, as suggested by e.g., Lucas [64], Romer [65], De Dominicis et al. [66], Sala-I-Martin et al. [67], among others, our study also uses measures of human capital (denoted as *hc*) and physical capital reflected by the share of gross capital formation (*csh*). Data on human capital (*hc*), physical capital (*csh*), and openness (*openness*) were taken from the PWT database.

Furthermore, to be consistent with empirical and theoretical studies of transition dynamics (see in [68]), we control for convergence using the natural logarithm of per capita income ln(y). Finally, we consider the recent results of Gundlach and Paldam [69], who argue that the long-term growth path is hump-shaped due to the nature of the transition process between traditional and modern steady-states. Therefore, to better control for convergence dynamics, we also experiment with the square of per capita income. PWT data covers the period from 1951 to 2017 (with fewer observations for some countries). Simultaneously, measure of financial development (denoted as *FD*) has been available since 1980. As a result, models controlling for financial development are used on limited samples.

## 5. Results

### 5.1. Demonstration of the Application of Genetic Algorithm

An evaluation of the suitability of the genetic algorithm for detecting level breaks and growth rate volatility is performed on synthetic data. To this end, we generate a synthetic time series of length 70, with the first 35 observations generated by a different underlying process than the last 35. Specifically, for each i=1,2,3,…,70 we draw a random number from the normal probability distribution with mean μ and standard deviation σ as in Equation (6).

(6) $$X_{i}∼\left\{\begin{array}{cc} N(\mu_{1},\sigma_{1}^{2}) & \;\mathrm{for}\;1\leq i\leq 35\\ N(\mu_{2},\sigma_{2}^{2}) & \;\mathrm{for}\;36\leq i\leq 70 \end{array}\right.$$

At the beginning, we set $\mu_{1}$ and $\mu_{2}$ to 0, $\sigma_{1}$ to 0.1, and $\sigma_{2}$ to 2.0. The result is a time series with a fixed mean and drastically different variability. We repeat the procedure ten times and denote the time series as *A*. In the next step, we reduce the differences in variability before and after the 35th observation by setting $\sigma_{1}$ to 0.8 and $\sigma_{2}$ to 2.0, and thus obtain a second time series, which we denote as *B*. Finally, to construct time series *C* we use the same standard deviation as in *B*, but change $\sigma_{1}$ and $\sigma_{2}$ by setting them to 2.0 and 0, respectively. The above procedure allows us to generate time series with a single structural break at the 36th observation, associated with a change in volatility (time series *A* and *B*) and a simultaneous change in mean and volatility (time series *C*).

**Figure 1:**
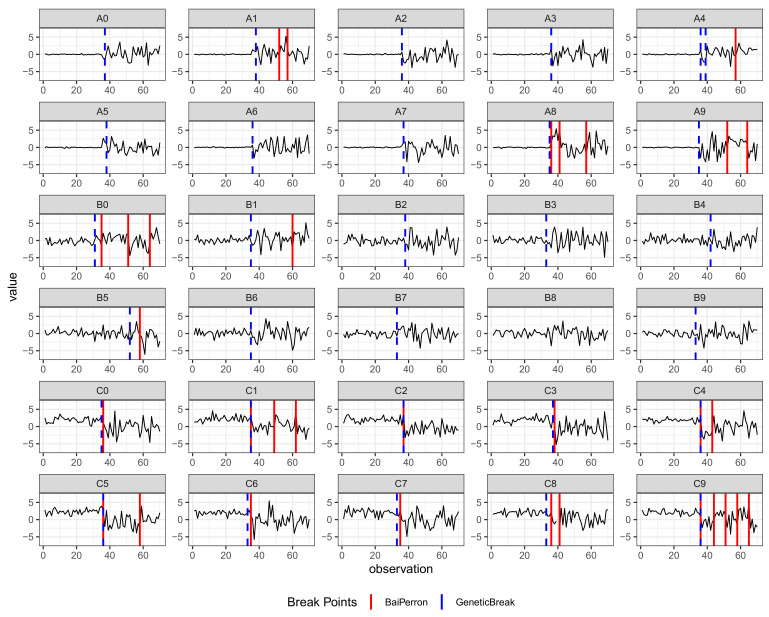
Synthetic time series generated as in Equation (6). Red vertical lines indicate structural breaks identified by Bai–Perron method. Blue vertical lines indicate structural breaks identified by the genetic algorithm. The true structural breaks in each time series are located at the 36th observation. GA outperforms BP in terms of fewer false positive and false negative breaks.

The breakpoints in the time series *A*, *B*, and *C*, detected by the Bai–Perron algorithm and the genetic algorithm, are shown in Figure 1. The results reveal clear differences in the results of the algorithms. The Bai–Perron procedure is unable to detect true breaks in the time series *A* and *B*. At the same time, the GA mostly points to a break at about the 36th observation. In the time series *C*, both GA and BP procedures correctly identify breaks around the 36th observation. At the same time, the Bai–Perron method gives some false positives for series with relatively high variability. For the genetic algorithm, this problem is almost absent. Consequently, the experiment suggests that GA is more suitable for separating short-term shocks from structural breaks for time series with high volatility. In addition, GA performs well in segmenting time series based on their volatility.

**Figure 2:**
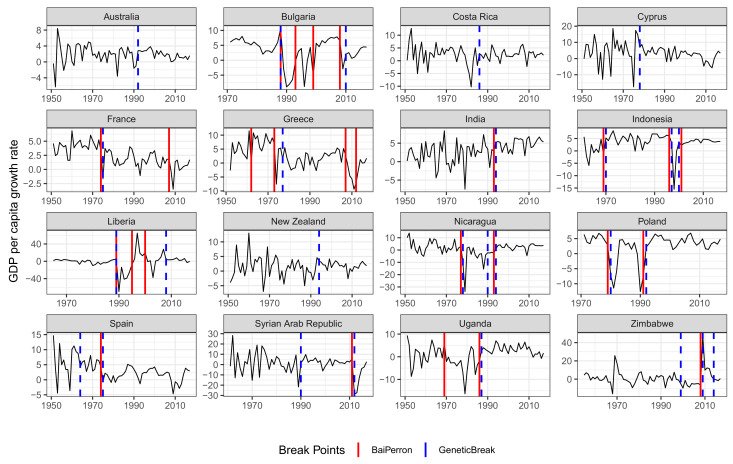
Growth rate of GDP per capita in selected countries. The red vertical line indicates structural breaks identified by the Bai–Perron method. The blue vertical line indicates structural breaks identified using the genetic algorithm.

Below, we present the GA results for the time series of GDP growth rates for a subset of countries. As in the previous case, we compare the results obtained using GA with those obtained using the Bai–Perron method, which were shown in Figure 2. The result of this comparison shows that the properties of GA described above have important implications in its application to the study of economic growth and its episodes. In particular, applying the genetic algorithm to time series of growth rates allows us to identify structural breaks that represent changes in the volatility of growth rates. The most prominent examples are Australia, Costa Rica, Cyprus, New Zealand, Spain, and the Syrian Arab Republic—in these cases, the genetic algorithm splits time series into episodes with fairly similar growth rates but with contrasting variability. Meanwhile, the BP method fails to identify any break in these series. Thus, we find a confirmation of the results obtained in experiments with artificial data. Simultaneously, Figure 2 clearly confirms that, in many cases, countries had episodes of volatile growth followed by episodes of stabilization.

### 5.2. Identification of Structural Breaks and Growth Episodes

**Figure A1:**
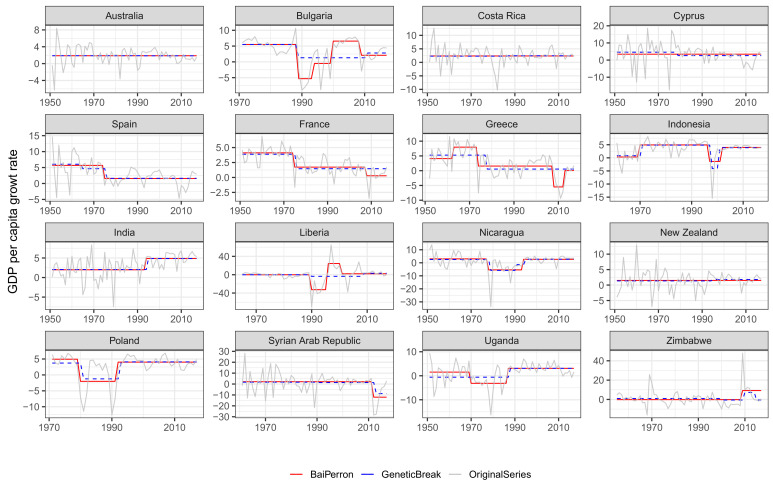
Average GDP growth rates within episodes in selected countries. Blue line presents average growth rates within episodes elicited with the genetic algorithm. Red lines stand for the average growth rates within episodes identified with the Bai–Perron method. Raw GDP growth time-series are presented with the gray lines.

Applying the GA procedure to the growth time series of each country in our sample allowed us to extract 371 growth episodes. For comparison purposes, we perform analogous calculations using the Bai–Perron technique, which in turn allows us to extract 394 segments. For each of these, we compute the average growth rate within an episode (see Figure A1 in the Appendix A for examples of the results of such computations). In the next step, we classify the resulting growth episodes according to the criteria presented in [48]. Table 1 shows summary statistics for the six categories of growth episodes characterized by the average growth rate (see Section 3).

It is worth emphasizing the differences between growth episodes identified by GA and BP techniques. Most episodes detected with GA have a relatively high standard deviation (except for episodes of rapid growth). Thus, using GA we were able to detect more episodes of volatile growth. Note that BP finds more growth collapses and growth miracles. Some of these are classified by GA as temporary shocks from the long-run path, so the categories of stagnation (0 to 2%) and moderate growth (2 to 4%) are more common under this procedure.

Having identified episodes of economic growth using GA and BP techniques, in the next step we examine the relationship between economic growth and its volatility. Accordingly, an ordinal logistic regression model (Equation (3)) is run, where the ordinal dependent variable is the economic growth episode category, according to Equation (2). The estimation results of ordinal logistic regression for episodes elicited by BP and GA techniques are presented in Table 2 and Table 3, respectively.

Note that both procedures yield a negative relationship between growth and volatility. Accordingly, our study shows that higher growth volatility is associated with a lower probability of a country achieving a more prosperous growth episode. The baseline results on the volatility-growth linkage are thus consistent with other studies, such as those in [21,22]. At the same time, estimates based on GA episodes, compared to BP episodes, indicate a stronger effect of volatility on growth. The results of both procedures are robust to the inclusion of control variables, including year and region dummies.

Regarding the control variables, we find evidence of positive relationships between economic growth and physical capital, human capital, and international trade. Moreover, the estimations using BP and GA techniques illustrate that the lower the level of development of a country, the higher the probability of entering a higher growth path, which reflects the convergence hypothesis. When the nonlinear dynamics of convergence are taken into account (Table 2 and Table 3, Models 4), the results indicate a hump-shaped convergence path, as suggested by the authors of [69]. Interestingly, the parameter of the FD variable suggests that deeper financial development corresponds to lower growth rates, with constant volatility. Such a result raises the conjecture that financial intermediation may promote growth through the stability channel while lowering growth through other mechanisms.

To test whether the results are affected only by the issue of growth collapses (which has the highest volatility), we exclude episodes with average growth rates below −2%. As expected, the coefficient of growth volatility is higher in such cases (see the last column of Table 3), but remains negative and significant. Thus, the negative relationship between these variables persists also in cases less extreme than growth collapses.

The estimation of Equation (4), which relaxes the proportional odds assumption embedded in the baseline model given by Equation (3), also yields interesting results. Here, we test whether the coefficient of volatility changes when the dependent variable is dichotomized based on different threshold levels of within-episode growth rates. Thus, the coefficient of volatility is interpreted as an estimate of the effect of growth instability on the odds of achieving a growth episode higher than a given threshold. As reported in Table 4, high volatility is negatively related to growth rate only up to a certain level. The estimates of Equation (4) illustrate that lower volatility is associated with growth episodes above −2%, above 0%, and above 2%, rather than with growth episodes below these thresholds. At the same time, volatility is not a significant predictor of a growth episode above the 4% threshold. Thus, despite the overall negative relationship between growth and volatility, the relationship remains vague for episodes of the highest growth rates. The most prosperous episodes appear to be driven by other, more fundamental factors of economic growth, such as the initial level of output and underlying convergence processes, as well as international trade and investment in physical capital.

The estimates presented in Table 4 also reveal some interesting insights into the relationship between controls and the probability of achieving growth above selected threshold levels. In particular, openness to international trade (*openness*) increases the probability of reaching a higher growth episode. However, this positive relationship weakens and eventually turns negative when the economy reaches an episode of rapid growth (above 6%). Significant nonlinearities also become apparent for financial development. The overall interpretation for all the models in Table 4 suggests that countries with relatively well-developed financial systems tend to stagnate (growth rates of 0% to 2%) or to grow at very high rates (above 6%). At the same time, these countries are less likely to experience growth collapses, periods of negative growth, but also periods of moderate and rapid growth.

### 5.3. Growth-Volatility Patterns

**Figure 3:**
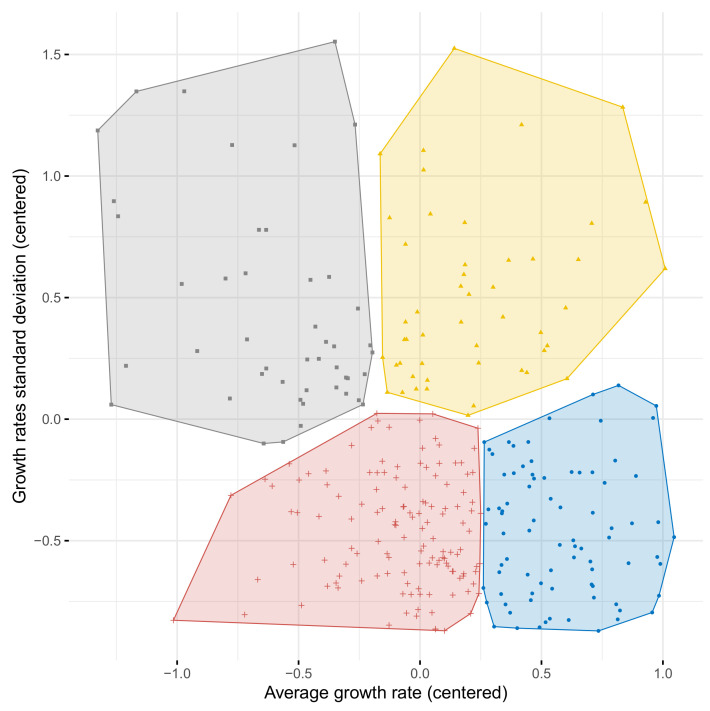
Within-episode average growth rate and standard deviation. Each symbol represents one episode. Episodes were obtained from inflection points detected by the generic algorithm. Clusters of episodes, extracted using the k-means algorithm, were represented by the colors and shapes of the symbols.

In this subsection, we turn to the study of factors that enhance stable and high growth. We begin by applying a k-means clustering procedure to identify groups of episodes with similar volatility and average growth rates. The k-means algorithm, applied to episodes elicited with the GA-based technique, returned the clusters shown in Figure 3.

**Figure A2:**
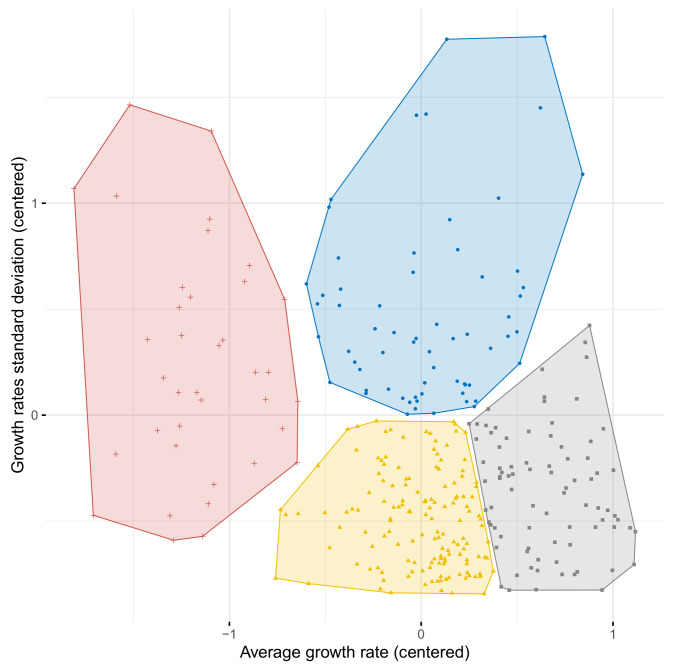
Within-episode average growth rate and standard deviation. Each symbol represents one episode. Episodes were obtained from inflection points detected by the Bai–Perron method. Clusters of episodes, extracted using the k-means algorithm, were represented by the colors and shapes of the symbols.

For ease of interpretation, we limited the number of clusters to four. As can be observed in the graph, the central region of the plot is filled with a dense cloud of points illustrating fairly similar episodes. Many episodes with volatility and growth rates close to the sample averages are separated into different clusters, despite the lack of clear distinction. Thus, the separation of the resulting clusters is not so obvious. Nevertheless, the k-means algorithm returns more compact clusters than the manual division of the sample based on thresholds set to sample means (the sum of squared distances within clusters is approximately 7% lower in the former case). Moreover, clusters of episodes elicited with GA turn out to be more informative and easier to interpret than clusters obtained for BP episodes (cf. Figure A2 in the Appendix A). Therefore, we use k-means clustering in our baseline model. To check for validity, we re-run our analysis for growth patterns separated by the sample averages for within-episode growth rate and within-episode growth standard deviation. Having identified clusters of episodes, we move on to analyze the determinants of class membership. In particular, we look for factors that increase the chances of following a development path characterized by an episode of high and stable economic growth. To this end, we estimated the parameters of the model given by Equation (5). Membership in one of the clusters shown in Figure 3 was taken as the dependent variable. The cluster in the bottom right corner of the plot is treated as a benchmark, so the coefficients shown in Table 5 and Table 6 should be interpreted in terms of the odds of following a given pattern versus a pattern of fast and stable growth. The analysis performed on the clusters identified using sample mean thresholds for within-episode growth and within-episode standard deviation is presented in the Appendix A, in Table A1. Because the results of this robustness check lead to similar conclusions, we will not discuss them in detail. Each of the estimated parameters of models 1–3 in Table 5 and models 1–2 in Table 6 are negative and significant for variables representing human capital accumulation *hc* and gross physical capital accumulation *csh_i*. Such results indicate that human and physical capital accumulation increases the chances of generating high and stable growth. Consequently, these results are consistent with the conventional belief that the acquisition of physical and human capital promotes economic growth [70,71,72]. Moreover, human and physical capital tend to smooth economic fluctuations. Thus, for these variables, we see no evidence of a trade-off between growth and stability. At the same time, we found that the lower the level of economic development, the more likely an episode of high and stable growth is, which is consistent with the neoclassical convergence hypothesis [68].

Another result from the estimation of the model given by Equation (5) concerns openness to international trade. The results reflect a positive correlation between openness and growth, i.e., countries with a higher share of trade in their GDP tend to achieve episodes of successful growth. This, in turn, comes with a stability cost as these countries become vulnerable to episodes of instability. These results are robust to different specifications in all our models. This finding corresponds to the works of Kose et al. [29], who claim that trade and financial integration appears to weaken the negative relationship between growth and volatility. In particular, they found that countries that are more open to trade seem to be able to tolerate higher volatility without negative consequences for long-term growth. Furthermore, we observe interesting results for financial development, which seems to promote stability rather than growth itself. Countries with advanced financial systems tend to follow paths of stable and low growth rather than fast and stable growth. At the same time, they are much less exposed to episodes of high volatility/high growth and less exposed to low growth and low volatility. The results obtained hold when we further control for episode duration and openness. Controlling for humped-shaped convergence paths makes the coefficient *FD* insignificant only for low and stable growth. In summary, we find confirmation of the results given by the model specified by Equation (3), which suggested that financial development is inversely correlated with dynamic growth with constant volatility. Thus, our results are consistent with the financialization literature [73,74], which casts doubt on the positive association between growth and financialization, from both heterodox and mainstream positions (see in [75]). At the same time, these results are consistent with Norrbin and Yigit [1], who claim that with the absence of financial intermediation, some countries—particularly developing countries—may be exposed to increased output volatility.

## 6. Discussion

This paper investigates the relationship between growth and volatility on a sample of 182 countries over the period 1951–2017. We used the growth episodes approach to find the link between growth and volatility.

Our key contribution to the literature is the identification of periods of growth stability. Specifically, we employed the genetic algorithm to identify the main breaks in volatility and average GDP growth, and segmented the GDP time series accordingly. In our view, there are two main advances resulting from such a procedure. First, it has paved the way for looking at the growth-volatility relationship from an episodic perspective. As postulated by Pritchett [2,76], such a perspective is crucial to properly capture the growth process. After identifying growth episodes, we analyzed the growth–variability relationship using ordinal logistic models. We found evidence of a negative relationship between volatility and growth. In particular, the results suggest that higher volatility is associated with a lower probability of moving to a more prosperous growth episode. Thus, our key findings are consistent with general theoretical settlements based on the Keynesian approach and endogenous growth theory, which emphasizes short-term losses associated with output fluctuations [17].

Second, the identification of growth segments with different levels of volatility opens the door to further inquiry into the forces responsible for generating a given pattern. In an attempt to explain low/high and stable/variable growth episodes, we used a multinomial regression model. Based on this, we found that physical and human capital correspond to episodes of stable growth. Thus, our findings are consistent with the theoretical and empirical literature on economic growth, which emphasizes a strong positive correlation between output growth and the acquisition of both human capital and physical capital, see, e.g., in [64,70]. Furthermore, our results support the hypothesis that openness to international trade stimulates rapid GDP growth, but at the expense of volatility. Specifically, we observed a positive relationship between openness and economic growth and a negative relationship between openness and growth stability. It is noteworthy that our finding of a positive relationship between openness to trade and economic growth and a negative one between openness and growth stability is consistent with research on the growth path of countries that have experienced economic crisis and recession since the early 1980s. In particular, openness to the world economy since the 1980s has been seen as an important determinant of high economic growth in developing and developed East Asian countries [77]. At the same time, excessive openness to the world economy has been blamed for macroeconomic instability and consequently greater volatility in the economic growth of these countries, which had devastating effects, particularly during the Asian financial crisis in the late 1990s [78].

Finally, we found that financial development increases stability but does not promote growth. This result may reflect the stabilizing effects of intermediation, as argued in [34], but also the negative links between capital accumulation and financialization, associated with “corporate short-termism” and shareholder orientation of publicly traded companies [75].

Consequently, our study supports the arguments raised by Pritchett [2]. In particular, our results indicate *implicitly* that both level of economic growth and its volatility are different in various segments of the GDP time series. Thus, in our study, we depart from an arbitrary setting of interval lengths of episodes of growth and its volatility, and therefore we are able to identify the mechanisms of shifting an economy between the recognized episodes of stable/unstable growth or stagnation. In that respect, our study overcomes some of the disadvantages typical of a traditional approach to the research on economic growth and its volatility.

Moreover, our research findings support the empirical settlements of scholars [1,21,22,23,24], who also provided evidence for the negative relationship between volatility and growth. Simultaneously, our results are consistent with studies that incorporate various economic phenomena into the relationship between the GDP growth and its volatility, such as e.g., in [29,30,32,33,34].

Having considered the main findings of our study, we can formulate several recommendations for economic policy. First, if the main goal of economic policy is sustained economic growth, then policy-makers should seek countercyclical policy measures that smooth the volatility of growth because, as we have shown, stability is associated with a higher probability of moving into an episode of more prosperous economic growth. Second, economic policy should be focused on raising both physical and human capital. As our study illustrates, the acquisition of physical and human capital supports greater output stability. Additionally, according to the conventional belief of economic growth literature, physical and human capital determine high economic growth in the long run. At the same time, our study shows that not all volatility reduction measures should be treated as growth-promoting. In particular, governments can expect less stability when foreign trade intensifies. Nevertheless, trade openness itself increases growth, even at the cost of higher volatility. Thus, promoting international trade should be seen as a growth-enhancing policy. In contrast, policies aimed at deepening financial development stabilize the economy but have limited potential to stimulate growth.

## Figures and Tables

**Table 1:** Summary statistics by growth episode.

Bai–Perron Episodes	n	Avg. sd	Avg. Growth	Avg. Duration
Below −2	69	8.2	−8.3	8.3
−2 to 0	39	4.8	−0.8	25.2
0 to 2	96	3.8	1.1	37.2
2 to 4	94	3.7	2.9	32.1
4 to 6	47	3.9	5.0	23.4
Over 6	49	6.2	9.6	11.7
**GA Episodes**				
Below −2	32	13.3	−8.3	9.3
−2 to 0	45	6.6	−0.8	23.9
0 to 2	117	4.3	1.2	32.9
2 to 4	102	3.8	2.9	30.5
4 to 6	49	3.4	4.9	23.7
Over 6	26	6.7	9.0	12.0

**Table 2:** Ordered logistic regression for growth episodes elicited with Bai–Perron.

	Model 1	Model 2	Model 3	Model 4
volatility	−0.10 ***	−0.11 ***	−0.10 ***	−0.07 ***
	(0.01)	(0.01)	(0.01)	(0.01)
log(y)	−0.33 ***	−0.56 ***	−0.63 ***	3.68 ***
	(0.03)	(0.04)	(0.05)	(0.32)
hc	0.37 ***	0.77 ***	0.11	−0.01
	(0.05)	(0.06)	(0.08)	(0.08)
csh_i	2.65 ***	3.75 ***	2.96 ***	2.70 ***
	(0.23)	(0.33)	(0.34)	(0.34)
openness	0.30 ***	0.42 ***	0.48 ***	0.57 ***
	(0.05)	(0.06)	(0.06)	(0.06)
FD		−0.58 **	−1.71 ***	−1.02 ***
		(0.19)	(0.21)	(0.21)
log(y${}^{2}$)				−0.24 ***
				(0.02)
Year and region dummies	No	No	Yes	Yes
AIC	23,688.97	14,521.98	13,623.78	13,429.73
BIC	23,758.86	14,593.68	13,969.25	13,781.71
Log Likelihood	−11,834.49	−7249.99	−6758.89	−6660.86
Deviance	23,668.97	14,499.98	13,517.78	13,321.73
Num. obs.	8014	5005	5005	5005

*** *p* < 0.001; ** *p* < 0.01; * *p* < 0.05.

**Table 3:** Ordered logistic regression for growth episodes elicited with the use of the genetic algorithm.

	Model 1	Model 2	Model 3	Model 4	Model 5
	Full Sample	Full Sample	Full Sample	Full Sample	Sample of Growth Above −2%
volatility	−0.18 ***	−0.27 ***	−0.27 ***	−0.26 ***	−0.14 ***
	(0.01)	(0.01)	(0.01)	(0.01)	(0.01)
log(y)	−0.44 ***	−0.58 ***	−0.59 ***	3.51 ***	−0.63 ***
	(0.03)	(0.04)	(0.05)	(0.32)	(0.05)
hc	0.75 ***	0.89 ***	0.31 ***	0.19 *	0.41 ***
	(0.05)	(0.06)	(0.08)	(0.08)	(0.08)
csh_i	3.13 ***	2.81 ***	2.04 ***	1.83 ***	2.78 ***
	(0.24)	(0.33)	(0.34)	(0.34)	(0.36)
openness	0.39 ***	0.60 ***	0.71 ***	0.78 ***	0.55 ***
	(0.05)	(0.06)	(0.06)	(0.06)	(0.06)
FD		0.13	−0.69 ***	−0.06	−0.77 ***
		(0.19)	(0.21)	(0.21)	(0.21)
log(y${}^{2}$)				−0.23 ***	
				(0.02)	
Year and region dummies	No	No	Yes	Yes	Yes
AIC	21,494.22	13,205.64	12,484.94	12,317.32	11,463.11
BIC	21,564.11	13,277.34	12,830.41	12,669.30	11,806.65
Log Likelihood	−10,737.11	−6591.82	−6189.47	−6104.66	−5678.55
Deviance	21,474.22	13,183.64	12,378.94	12,209.32	11,357.11
Num. obs.	8014	5005	5005	5005	4827

*** *p* < 0.001; ** *p* < 0.01; * *p* < 0.05.

**Table 4:** Logistic regression of growth episodes elicited using the genetic algorithm.

	Above −2	Above 0	Above 2	Above 4	Above 6
(Intercept)	8.57 ***	7.28 ***	3.92 ***	1.35 *	9.73 ***
	(1.14)	(0.60)	(0.47)	(0.60)	(2.14)
volatility	−0.44 ***	−0.28 ***	−0.18 ***	−0.00	0.02
	(0.03)	(0.02)	(0.02)	(0.01)	(0.03)
log(y)	−0.42 **	−0.95 ***	−0.46 ***	−0.48 ***	−1.71 ***
	(0.13)	(0.07)	(0.06)	(0.07)	(0.28)
hc	−0.54	1.46 ***	−0.06	0.89 ***	−0.55
	(0.40)	(0.17)	(0.10)	(0.13)	(0.31)
csh_i	−0.71	1.80 ***	3.45 ***	2.77 ***	10.90 ***
	(1.15)	(0.54)	(0.43)	(0.56)	(1.54)
openness	2.99 ***	0.47 *	1.07 ***	0.60 ***	−8.06 ***
	(0.52)	(0.18)	(0.10)	(0.09)	(1.12)
FD	5.20 ***	6.10 ***	−1.36 ***	−2.93 ***	3.93 ***
	(1.14)	(0.56)	(0.25)	(0.33)	(1.04)
Year and region dummies	Yes	Yes	Yes	Yes	Yes
AIC	774.02	2958.17	5651.30	3170.54	724.74
BIC	1093.42	3277.57	5970.69	3489.93	1044.13
Log Likelihood	−338.01	−1430.09	−2776.65	−1536.27	−313.37
Deviance	676.02	2860.17	5553.30	3072.54	626.74
Num. obs.	5005	5005	5005	5005	5005

*** *p* < 0.001; ** *p* < 0.01; * *p* < 0.05.

**Table 5:** Multinomial regression of GA growth episodes, part 1. Growth episodes clustered by the k-means algorithm.

	Model 1	Model 2	Model 3
High growth, high sd: log(y)	0.42(0.07) ***	1.09(0.11) ***	0.92(0.11) ***
Low growth, high sd: log(y)	1.55(0.08) ***	1.62(0.10) ***	1.76(0.10) ***
Low growth, low sd: log(y)	0.95(0.06) ***	0.96(0.07) ***	1.03(0.08) ***
High growth, high sd: hc	−1.40(0.13) ***	−1.21(0.19) ***	−1.46(0.21) ***
Low growth, high sd: hc	−2.58(0.13) ***	−2.87(0.18) ***	−2.73(0.19) ***
Low growth, low sd: hc	−0.66(0.08) ***	−1.34(0.11) ***	−1.31(0.11) ***
High growth, high sd: csh_i	−6.11(0.53) ***	−11.35(0.89) ***	−12.61(0.92) ***
Low growth, high sd: csh_i	−2.46(0.48) ***	−6.60(0.68) ***	−6.00(0.70) ***
Low growth, low sd: csh_i	−4.29(0.37) ***	−6.24(0.50) ***	−5.68(0.51) ***
High growth, high sd: duration	−0.18(0.05) ***	0.43(0.08) ***	0.42(0.08) ***
Low growth, high sd: duration	−0.55(0.06) ***	−0.34(0.08) ***	−0.33(0.08) ***
Low growth, low sd: duration	0.37(0.04) ***	0.23(0.05) ***	0.27(0.05) ***
High growth, high sd: openness	0.35(0.10) ***		1.41(0.15) ***
Low growth, high sd: openness	−1.55(0.18) ***		−1.42(0.24) ***
Low growth, low sd: openness	−0.56(0.07) ***		−0.57(0.09) ***
High growth, high sd: FD		−4.46(0.63) ***	−5.58(0.66) ***
Low growth, high sd: FD		−3.34(0.54) ***	−2.78(0.56) ***
Low growth, low sd: FD		1.22(0.30) ***	1.44(0.30) ***
Region dummies	Yes	Yes	Yes
AIC	14,360.05	8530.82	8339.07
BIC	14,609.92	8763.84	8591.51
Log Likelihood	−7144.02	−4229.41	−4130.54
Deviance	14,288.05	8458.82	8261.07
Num. obs.	7637	4783	4783
K	4	4	4

*** *p* < 0.001; ** *p* < 0.01; * *p* < 0.05.

**Table 6:** Multinomial regression of GA growth episodes, part 2. Growth episodes clustered by the k-means algorithm.

	Model 1	Model 2
High growth, high sd: log(y)	1.00(0.11) ***	−9.30(0.40) ***
High growth, high sd: log(y${}^{2}$)		0.60(0.02) ***
Low growth, high sd: log(y)	1.79(0.10) ***	−12.04(0.37) ***
Low growth, high sd: log(y${}^{2}$)		0.80(0.02) ***
Low growth, low sd: log(y)	1.10(0.08) ***	−7.83(0.31) ***
Low growth, low sd: log(y${}^{2}$)		0.52(0.02) ***
High growth, high sd: hc	−1.68(0.20) ***	−1.16(0.21) ***
Low growth, high sd: hc	−2.63(0.18) ***	−2.42(0.20) ***
Low growth, low sd: hc	−1.47(0.11) ***	−1.13(0.12) ***
High growth, high sd: csh_i	−12.19(0.90) ***	−12.18(0.94) ***
Low growth, high sd: csh_i	−6.47(0.71) ***	−5.38(0.74) ***
Low growth, low sd: csh_i	−5.59(0.51) ***	−5.08(0.53) ***
High growth, high sd: openness	1.40(0.15) ***	1.19(0.15) ***
Low growth, high sd: openness	−1.34(0.23) ***	−2.00(0.27) ***
Low growth, low sd: openness	−0.54(0.09) ***	−0.83(0.09) ***
High growth, high sd: FD	−4.75(0.63) ***	−8.17(0.70) ***
Low growth, high sd: FD	−3.38(0.54) ***	−5.96(0.63) ***
Low growth, low sd: FD	1.84(0.30) ***	−0.50(0.35)
High growth, high sd: duration		0.49(0.08) ***
Low growth, high sd: duration		−0.27(0.08) ***
Low growth, low sd: duration		0.29(0.05) ***
Region dummies	Yes	Yes
AIC	8445.00	7985.22
BIC	8678.02	8257.08
Log Likelihood	−4186.50	−3950.61
Deviance	8373.00	7901.22
Num. obs.	4783	4783
K	4	4

*** *p* < 0.001; ** *p* < 0.01; * *p* < 0.05.

## Data Availability

Some of the data used was synthesised according to a precisely described set of rules. Remaining data is available in a publicly accessible repository, the Penn World Table (PWT) database version 9.1 [59].

## Appendix A

**Table A1:** Multinomial regression of GA growth episodes. Growth patterns separated by sample averages of within-episode growth rate and within-episode standard deviation.

	Model 1	Model 2	Model 3	Model 4
High growth, high sd: log(y)	0.18(0.07) *	0.98(0.10) ***	0.90(0.10) ***	−2.22(0.29) ***
High growth, high sd: log(y${}^{2}$)				0.18(0.02) ***
Low growth, high sd: log(y)	0.90(0.06) ***	1.08(0.08) ***	1.06(0.08) ***	−5.24(0.43) ***
Low growth, high sd: log(y${}^{2}$)				0.36(0.02) ***
Low growth, low sd: log(y)	1.01(0.06) ***	0.98(0.07) ***	1.03(0.07) ***	−1.71(0.42) ***
Low growth, low sd: log(y${}^{2}$)				0.15(0.02) ***
High growth, high sd: hc	−1.64(0.12) ***	−0.41(0.19) *	−0.57(0.20) **	−0.45(0.20) *
Low growth, high sd: hc	−2.02(0.11) ***	−1.99(0.15) ***	−1.98(0.15) ***	−1.78(0.16) ***
Low growth, low sd: hc	−1.26(0.09) ***	−1.79(0.12) ***	−1.71(0.12) ***	−1.63(0.12) ***
High growth, high sd: csh_i	−3.22(0.49) ***	−5.00(0.87) ***	−5.39(0.88) ***	−5.19(0.88) ***
Low growth, high sd: csh_i	−3.30(0.42) ***	−5.19(0.58) ***	−5.13(0.59) ***	−4.97(0.61) ***
Low growth, low sd: csh_i	−4.71(0.41) ***	−4.18(0.51) ***	−3.76(0.52) ***	−3.54(0.52) ***
High growth, high sd: duration	−0.72(0.05) ***	0.32(0.09) ***	0.30(0.09) ***	0.32(0.09) ***
Low growth, high sd: duration	−0.61(0.05) ***	−0.27(0.06) ***	−0.26(0.06) ***	−0.19(0.06) **
Low growth, low sd: duration	−0.61(0.04) ***	−0.54(0.05) ***	−0.53(0.05) ***	−0.51(0.05) ***
High growth, high sd: openness	0.37(0.10) ***		0.77(0.18) ***	0.68(0.19) ***
Low growth, high sd: openness	−0.47(0.13) ***		0.10(0.16)	0.00(0.17)
Low growth, low sd: openness	−0.44(0.10) ***		−0.63(0.13) ***	−0.72(0.13) ***
High growth, high sd: FD		−8.57(0.64) ***	−9.08(0.66) ***	−9.77(0.68) ***
Low growth, high sd: FD		−4.16(0.46) ***	−4.24(0.47) ***	−5.72(0.51) ***
Low growth, low sd: FD		0.88(0.32) **	1.08(0.32) ***	0.70(0.34) *
Region dummies	Yes	Yes	Yes	
AIC	14,086.88	8454.27	8408.75	8261.68
BIC	14,336.75	8687.29	8661.19	8533.54
Log Likelihood	−7007.44	−4191.13	−4165.37	−4088.84
Deviance	14,014.88	8382.27	8330.75	8177.68
Num. obs.	7637	4783	4783	4783
K	4	4	4	4

*** *p* < 0.001; ** *p* < 0.01; * *p* < 0.05.
